# Needs assessment for enhancing pediatric clerkship readiness

**DOI:** 10.1186/s12909-023-04167-7

**Published:** 2023-03-28

**Authors:** Adam Weinstein, Peter MacPherson, Suzanne Schmidt, Elizabeth Van Opstal, Erica Chou, Mark Pogemiller, Kathleen Gibbs, Melissa Held

**Affiliations:** 1 Frank H. Netter MD School of Medicine, North Haven, CT USA; 2 Dalhousie University, Nova Scotia, Canada; 3 Northwestern University Feinberg School of Medicine, Chicago, IL USA; 4 Rush University, Chicago, IL USA; 5 Medical College of Wisconsin, Milwaukee, WI USA; 6 University of Oklahoma, Oklahoma City, OK USA; 7 University of Pennsylvania, Philadelphia, PA USA; 8 University of Connecticut School of Medicine, Farmington, CT USA

**Keywords:** Pediatric clerkship, Pediatric physical examination, Clinical skills

## Abstract

**Background:**

Many students report feeling inadequately prepared for their clinical experiences in pediatrics. There is striking variability on how pediatric clinical skills are taught in pre-clerkship curricula.

**Methods:**

We asked students who completed their clerkships in pediatrics, family medicine, surgery, obstetrics-gynecology and internal medicine to rate their pre-clinical training in preparing them for each clerkship, specifically asking about medical knowledge, communication, and physical exam skills. Based on these results, we surveyed pediatric clerkship and clinical skills course directors at North American medical schools to describe the competence students should have in the pediatric physical exam prior to their pediatric clerkship.

**Results:**

Close to 1/3 of students reported not feeling adequately prepared for their pediatrics, obstetrics-gynecology, or surgery clerkship. Students felt less prepared to perform pediatric physical exam skills compared to physical exam skills in all other clerkships. Pediatric clerkship directors and clinical skills course directors felt students should have knowledge of and some ability to perform a wide spectrum of physical exam skills on children. There were no differences between the two groups except that clinical skills educators identified a slightly higher expected competence for development assessment skills compared to pediatric clerkship directors.

**Conclusions:**

As medical schools undergo cycles of curricular reform, it may be beneficial to integrate more pre-clerkship exposure to pediatric topics and skills. Further exploration and collaboration establishing how and when to incorporate this learning could serve as a starting point for curricular improvements, with evaluation of effects on student experience and performance. A challenge is identifying infants and children for physical exam skills practice.

**Supplementary Information:**

The online version contains supplementary material available at 10.1186/s12909-023-04167-7.

## Background

Early clinical exposure and clinical skill development in medical school training is important for students to be successful during clerkships. Inadequate preparation may impact students’ ability to learn key skills, knowledge, and behaviors. A study examining clerkship directors’ views regarding pre-clerkship preparation in core clinical competencies found that 80% of clerkship directors felt that proficiency is needed in communication skills, professionalism, and interviewing/physical exam skills before entering the clerkships [1].

Historically, medical schools have taught a broad overview of basic sciences and clinical skills during the pre-clinical curricula. There is striking variability on how and when pediatric content and skills are taught to medical students [2]. In a previously published study, we found that more than one third of 3^rd^ year medical students completing their pediatric clerkship at one of four U.S. medical schools did not feel well-prepared for the clerkship [3]. In addition, one third of students specifically felt unprepared to perform pediatric physical exam skills. However, feeling unprepared is not limited to the pediatric clerkship [4–8]. In qualitative studies, students report struggling with several tasks, including “understanding roles and responsibilities, adjusting to clinical cultures, performing clinical skills, learning the logistics of clinical settings, and encountering frequent changes in staff, settings, and content.” In addition to these challenges, clerkship directors also noted that students had difficulties applying their medical knowledge to clinical scenarios, demonstrating clinical reasoning, and being self-directed learners [5]. In a quantitative assessment, Wenrich et al. compared the expectations of pre-clinical faculty, clerkship faculty, and third year medical students regarding clinical skills preparation for clerkships at a single North American medical school. Students had higher expectations than clerkship faculty for both basic and advanced clinical skills, as well as higher expectations than pre-clinical faculty for advanced clinical skills. Additionally, for many basic clinical skills, pre-clinical faculty had higher expectations of students compared to clerkship faculty [8].

As medical schools undergo curricular reform, there are few data to guide what content or skills training should be included in the pre-clinical arena. Although previously published data showed that students did not feel well prepared for the pediatric clerkship including the competencies of knowledge, communication, and physical exam skills, we felt it was important to distinguish whether this applied to pediatrics in particular, or if students feel unprepared for clerkships in general. This study aims to compare how students perceive their preparedness for each of their clinical clerkships. Additionally, based on student responses regarding decreased preparedness in physical exam skills, we explored this further by surveying pediatric clerkship directors and clinical skills curriculum directors. We asked these two groups of educators representing the curricular experts who typically lead instruction on physical exam skills and pediatric clinical skills, assessing the expected competency levels of students’ pediatric physical exam skills prior to starting the pediatric clerkship.

## Methods

This study was a product of the Pre-clinical and Clinical Skills Collaborative of the Council on Medical Student Education in Pediatrics (COMSEP). As part of the development.process for a standard pre-clinical pediatric curriculum, the authors reviewed the literature to identify pediatric physical exam skills as a particular area for study.

### Medical student clerkship preparedness survey development

The survey was developed using previously published methods [3]. Students were asked to “rate how the training in the first two years of medical school prepared them for: 1) the clerkship overall, 2) the communication skills needed for the clerkship, 3) the physical exam skills needed for the clerkship, and 4) the medical knowledge needed for this clerkship.” The survey used a 1–5 Likert (1 = poor, 5 = excellent).

The clerkship directors in pediatrics, internal medicine, and obstetrics-gynecology at three participating institutions, and for family medicine and general surgery at two of these institutions, distributed the survey to all medical students from November 2013 to February 2015. All three participating institutions have current LCME accreditation; one is in a rural setting, one in a suburban/small city and one in a major city. All three schools had traditional curricular structures with a two-year pre-clinical phase followed by a two-year clinical education phase. Over the study period, School A had 120 medical students complete the third year, School B had 112 students and School C had 75 students. All surveys were administered on the last day of the rotation either as part of the electronic final evaluation of the rotation or a paper survey, per the school’s customary process for end-of-clerkship block student evaluation. All responses were anonymous and voluntary, and there were no reminders or incentives. A total of 307 students across three medical schools were surveyed during the study period. Due to the timing of the study and variability in student clerkship schedules, not all students completed all clerkships during the study period, and the family medicine and surgery surveys were only conducted at two of the three schools. This led to a different total number of students surveyed for each type of clerkship. IRB approval was obtained at each institution.

### Pediatric and clinical skills educator physical exam survey development

A questionnaire was designed for pediatric clerkship and clinical skills curriculum directors to serve as a needs assessment on their perceptions of the competence expected of students in different components of the physical exam in pediatric patients, prior to the start of their pediatric clerkship. The survey was piloted by the authors, and subsequently by separate survey review teams of COMSEP, and of the Directors of Clinical Skills Education (DOCS). After incorporating recommendations from each of these teams, the survey was finalized and distributed to the listservs and member lists of COMSEP, representing 504 pediatric clerkship educators in the United States and Canada, and DOCS, representing 270 clinical skills educators (including a wide variety of medical specialties) in the United States and Canada at the time of distribution. (Supplemental Fig. 1) We specifically invited the subset of these educators who are pediatric clerkship directors and clinical skills course directors, respectively, to complete the survey. Additionally, the first question of the survey asked participants to identify their role in medical student education. Only the responses of those who identified as a pediatric clerkship director or clinical skills course director were included in our study, and the responses of those who serve as other medical student educators were excluded. These educators were asked “What level of competence should medical students have in performing pediatric specific physical exam skills on the day they start their pediatric clerkship” for each of the organ system exams specified, as well as the newborn exam and development assessment skills. The rating scale ranged from “no knowledge of exam maneuver” to “ability to perform maneuver.” They were also asked, and to select all that apply, “When are pediatric specific exam skills taught for all students in your school” and “When should pediatric specific exam skills be taught for all students in your school.” Ethics approval for the survey of pediatrics clerkship and clinical skills directors was obtained at Queen’s University’s Health Sciences & Affiliated Teaching Hospitals Research Ethics Board (HSREB).

### Analysis

Student survey responses were analyzed using Chi-Squared comparing total Fair-Poor responses (“inadequately prepared”) to total Good-Very Good–Excellent (“well prepared”) responses. Faculty survey responses were analyzed with paired t-test. *p* < 0.05 was considered significant.

## Results

### Medical students clerkship preparedness survey

There were 97 student responses (42% response rate) from the three schools for obstetrics-gynecology, 229 responses for pediatrics (97% response rate), 208 responses for internal medicine (74% response rate), 73 for family medicine (45% response rate), and 63 for surgery (38% response rate). Each clerkship specialty had a different number of students surveyed, as students’ schedules varied within and among schools over the study duration, which included portions of 2 academic years, and the family medicine and surgery surveys were only conducted at two schools. Overall, thirty-three percent of respondents felt their pre-clinical education was “poor” or “fair” in preparing them for their pediatric clerkship. This compared to 4% for their family medicine clerkship, 11% for internal medicine, 30% for surgery, and 31% for their obstetrics-gynecology rotation (Table 1).

**Table 1:** Comparison of student perceptions of feeling prepared for each of their clerkships, overall

Overall Clerkship Preparation (# of total responses)	Percent Responding “Poor” or “Fair”	Percent Responding “Good,” “Very Good,” or “Excellent”
Pediatrics (229)	33%	67%
Internal Medicine (208)	11%	89%
Obstetrics-Gynecology (97)	31%	69%
Family Medicine (73)	4%	96%
Surgery (63)	30%	70%

**Fig. 1:**
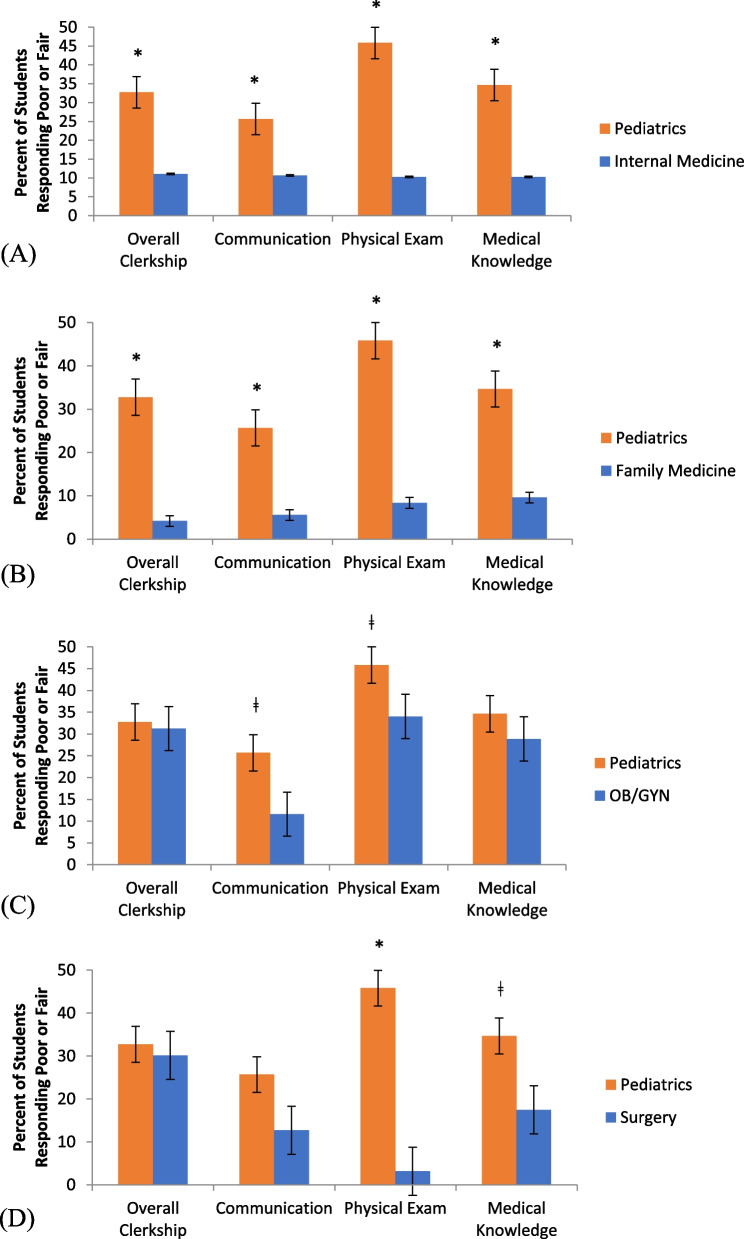
Comparison of student perceptions of feeling prepared between pediatrics and internal medicine (**A**), family medicine (**B**), obstetrics-gynecology (**C**), and surgery (**D**) clerkships. (ǂ *p* < 0.05 and * *p* < 0.005)

When comparing the percent of student responses of “fair or “poor” from pediatrics with other core clerkships, there were statistically significant differences in all areas assessed (overall, communication skills, physical exam skills and medical knowledge) for pediatrics compared to both internal medicine and family medicine (*p* < 0.005). For the physical exam skills, there were statistically significant higher numbers of “fair” and “poor” responses for pediatrics compared to all other core clerkships surveyed (*p* < 0.05) (Fig. 1).

### Pediatric and clinical skills educator physical exam survey

**Fig. 2:**
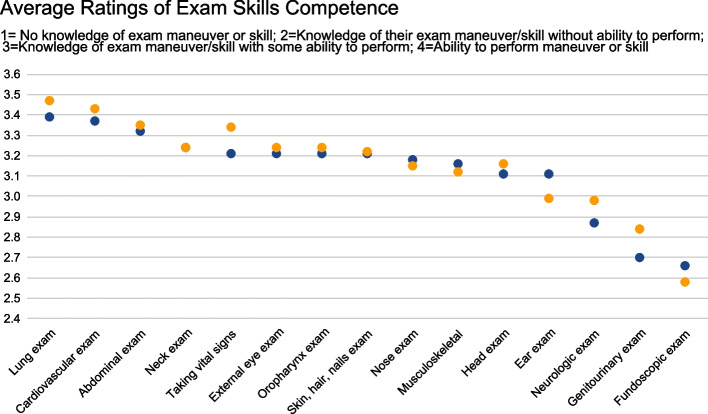
Level of competence medical students should have in performing pediatric specific physical exam skills on the day they start their pediatric clerkship. (Blue—Clinical Skills Directors; Orange—Pediatric Clerkship Directors)

**Fig. 3:**
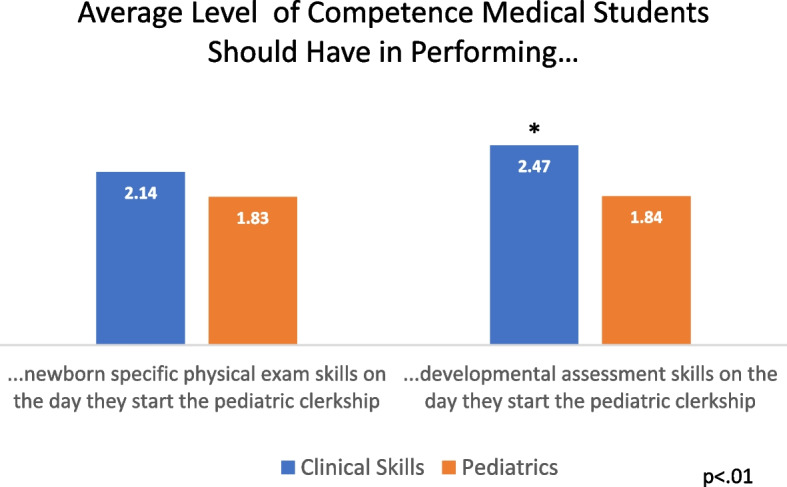
Level of competence medical students should have in performing newborn specific physical exam skills and development assessment skills on the day they start their pediatric clerkship. 1 = No knowledge of exam maneuver or skill; 2 = Knowledge of their exam maneuver/skill without ability to perform; 3 = Knowledge of exam maneuver/skill with some ability to perform; 4 = Ability to perform maneuver or skill

A total of 44 Clinical Skills Course directors and 91 Pediatric Clerkship directors completed the survey. For the majority of organ system physical exams, both clinical skills and pediatric educators felt students should have both knowledge of and some ability to perform the exam maneuvers on pediatric patients prior to the start of their pediatric clerkship (Fig. 2). Greater competence was expected by both groups of educators for the lung, cardiovascular, and abdominal exams, in which many educators felt students should have the full ability to perform these exams. With respect to the fundoscopic and genitourinary exam, many educators felt a lower competence was needed and that only knowledge of the exam in children was needed rather than some ability to perform the exam. There were no significant differences between pediatric clerkship and clinical skills directors. With respect to the newborn specific physical exam and childhood development assessment skills, both pediatric and clinical skills educators felt students should have some knowledge of the physical exam prior to their pediatric clerkship, but that students did not need to demonstrate an ability to perform the skill pre-clerkship (Fig. 3). Notably, there was a statistically significant difference in that more clinical skills directors felt that students ought to have some ability to perform development assessment skills as compared to pediatric clerkship directors (*p* < 0.0001).

**Fig. 4:**
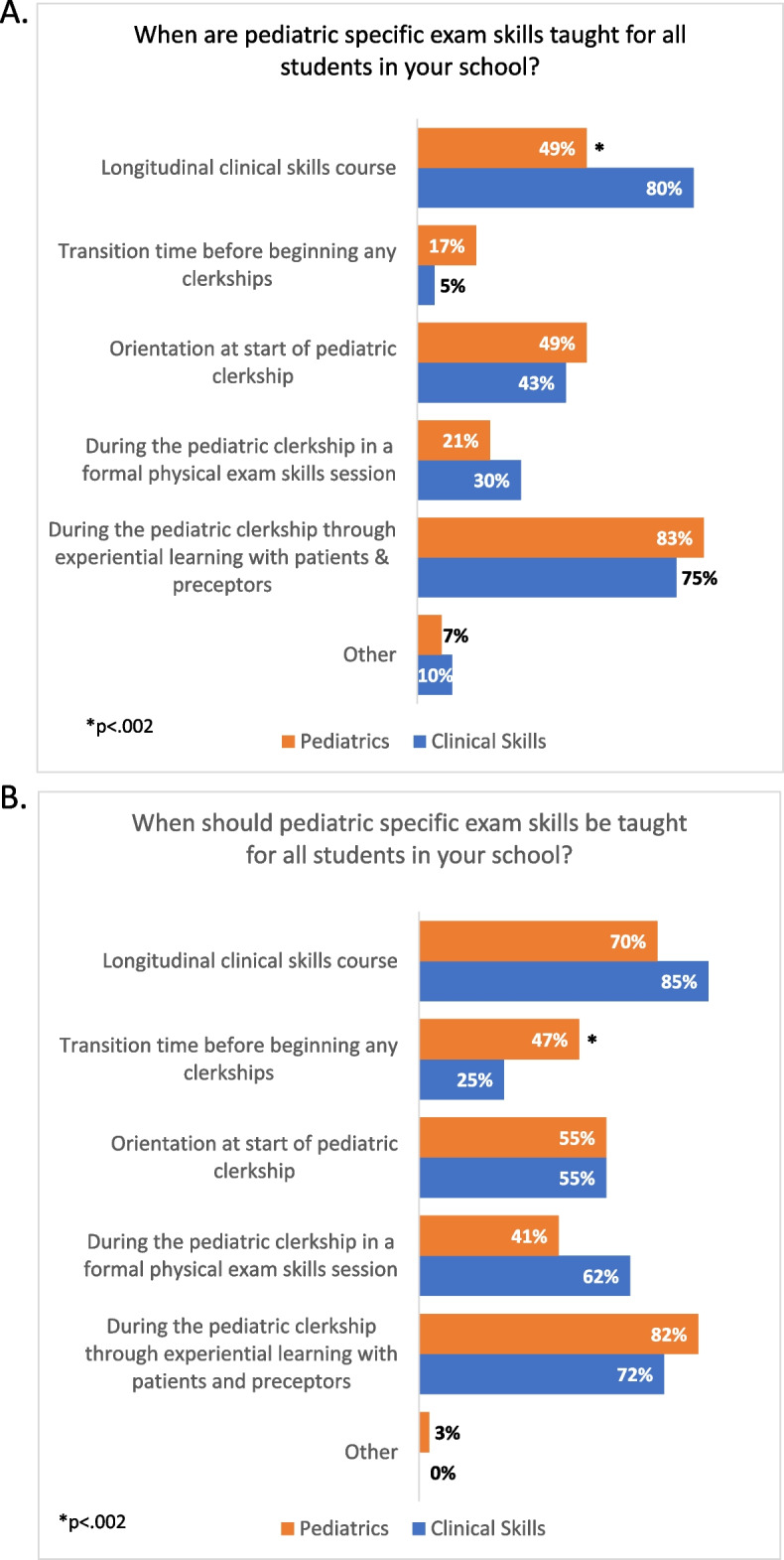
When clinical skills directors and pediatric clerkship directors believe pediatric specific exam skills *are* taught for students in their school (**A**), and when they believe they *should be* taught for students in their school (**B**)

We asked both groups when pediatric specific exam skills are taught for all students in their school, as well as when they *should* be taught for all students in their school (Fig. 4). Most clinical skills directors (80%) felt that pediatric specific exam skills were taught in the pre-clinical clinical skills course, whereas only 49% of pediatric clerkship directors identified this (*p* < 0.002). On the other hand, both groups felt the longitudinal clinical skills course was a curriculum where these skills should be taught (85% of clinical skills course directors and 70% of pediatric clerkship directors, no significant differences). Of note, the minority of both sets of educators (17% of pediatric clerkship, 5% of clinical skills directors) identified a transition time or orientation before beginning any clerkships as a place where these skills were being taught, but close to half of pediatric clerkship directors (47%) thought this would be an appropriate time compared to 25% of clinical skills directors (*p* < 0.002). Both groups felt pediatric physical exam skills should continue to be taught during the pediatric clerkship through a mixture of a skills session during clerkship orientation or during the clerkship, and through experiential learning with patients and preceptors.

## Discussion

To our knowledge, this is the first study to compare student perceptions of preparedness for their pediatric clerkship to other core clerkships based on their pre-clinical training. Close to one-third of medical students felt their pre-clinical education did not prepare them well for their pediatric clerkship overall. In the areas of communication skills, physical exam, and medical knowledge, medical students felt less prepared for pediatrics compared to family medicine and internal medicine. This may reflect the focus on adult medicine in the areas of medical knowledge, clinical skills, and communication skills that are typically taught in the early medical school curricula. Medical students specifically reported feeling less prepared for pediatric physical exam skills compared to all other specialties surveyed.

A similar number of students felt “unprepared” overall for pediatrics, obstetrics-gynecology and surgery. This is not surprising since a substantial component of learning for the medical student in obstetrics-gynecology and surgery relates to the operating room and surgical skills. Assisting in the labor and delivery suite is also a novel experience for most students [9]. The surgical literature also notes that a lack of any exposure to surgical skills in pre-clinical education can negatively impact students in various career choices. Exposure to select skills prior to the clerkship experiences may have a positive effect on early skill acquisition and enable more time-efficient training in the clerkship in an era where there is compression of clerkship clinical experiences [10].

In 2005, the Association of American Medical Colleges (AAMC) released a report titled “Recommendations for Clinical Skills Curricula for Undergraduate Medical Education.” In this report, the committee stated that the competencies for undergraduate medical education should provide fundamental clinical competencies that provide the foundation for later and more sophisticated levels of clinical practice [11]. One recommendation in particular states that students should demonstrate “The ability to provide clinical care within the practical context of a patient’s age, gender, personal preferences, family, health literacy, culture, religious perspective, and their economic circumstances.”

Some may feel that most pediatric-specific physical exam skills can be taught during the actual clerkship and do not need significant pre-clerkship attention. However, as the duration of clerkships has continued to shorten over the past decade, the time to develop these experiences within the context of the clerkship becomes more challenging. According to the AAMC, the average number of weeks for the pediatric clerkship at LCME accredited medical schools has declined, from 7.4 weeks in 2010–2011 to 6.6 weeks in 2018–2019 (https://www.aamc.org/data-reports/curriculum-reports/report/curriculum-reports). In response to this and with ongoing limited resources, some schools have gone to a longitudinal integrated clerkship approach with favorable results [12]. This application as a way to improve overall clinical skills, knowledge and overall experience in specialties like pediatrics, obstetrics-gynecology or general surgery is unclear but suggests further study.

When comparing the expectations of pediatric clerkship directors and clinical skills course directors regarding pediatric physical exam skill preparation, we found that for a wide array of organ system physical exam skills, pediatric clerkship directors and clinical skills course directors agreed that students should have knowledge of and some ability to perform the physical exam on children. On the other hand, both sets of directors felt that students ought to have knowledge of the newborn specific exam and developmental assessment skills, but on average did not feel it was necessary for them to have ability to perform these skills prior to the pediatrics clerkship. Notably, clinical skills directors believed pre-clinical students should demonstrate more competence in these areas as compared to pediatric clerkship directors. These differences may relate to their contexts in clinical education, where clinical skills educators aim for a certain level of comprehensive competence across many disciplines in their curricula, pediatric clerkship directors could be identifying specific skills achieved particularly well in the context of their specialty. Of note, Wenrich et al. also found that pre-clinical faculty had higher expectations of students’ preparedness in advanced clinical skills compared to clerkship directors [8]. These differences in expectations, as well as those of third year medical students, could be an area for future exploration.

Interestingly, only ~ 50% of pediatric clerkship directors felt that pediatric clinical skills were being taught in longitudinal clinical skills courses, as compared to ~ 80% of clinical skills course directors. Meanwhile, 70 and 85%, respectively, believe these skills should be taught in the pre-clinical clinical skills course. This discrepancy could be related to previously observed challenges in integrating and collaboration among pre-clinical and clinical curricular leaders. Mechanisms for collaborative sharing and curricular integration may reconcile this [13, 14]. Additionally, about half of pediatric clerkship directors believe pediatric physical exam skills ought to be taught in a transition to clerkship curriculum, whereas only ~ 25% of clinical skills course directors feel that way. If these skills can be successfully integrated into the longitudinal clinical skills course, then this timing could serve as an efficient opportunity for spaced learning and deliberately reinforcing these skills in a context closer to the pediatric clerkship. Both sets of educators identify the pediatric clerkship itself as an optimal context for pediatric physical exam skills education, recognizing that certain exams and achievement of greater competence to perform these skills is best achievable in the clinical specialty setting.

An intriguing future direction could be a collaboration among clinical skills and pediatric medical student educators on a shared pre-clinical pediatric physical exam skills curriculum with an evaluation of the student experience and student performance by students and faculty. Since both sets of educators value this experience, a major challenge could be finding and prioritizing curricular time, especially given trends over the last decade for schools to shorten the duration of their pre-clinical curriculum (https://www.aamc.org/data-reports/curriculum-reports/report/curriculum-reports). Another major challenge remains identifying children for medical student physical exam skills education. Different approaches to this challenge may work best at different medical schools. Among the possible solutions include scheduled pre-clinical experiences at pediatric outpatient, inpatient and/or pediatric specialty sites with goals for students to learn and practice physical exam skills under supervision by pediatric providers [15]. Another opportunity could be through collaboration with local daycare and school settings, where medical students could provide service and volunteer support (e.g. at school based clinics, vaccine clinics, health screenings sessions), while gaining opportunity to work with children of different ages and practice supervised physical exams. Simulated experiences provide a rich opportunity for students to learn and practice clinical skills on adult simulated patients. Creating analogous simulated opportunities with assented children could be another strategy.

There are several limitations to our study. Only three medical schools participated in the student survey. Schools were well sampled as far as size and location (city vs. rural) but all schools are geographically in the northeast part of the United States. The student survey results are also based on student perception and do not necessarily reflect student ability. We also only surveyed at schools who have traditional curricula with two pre-clinical education years followed by two clinical education years. With many schools adjusting to less time in the pre-clinical curriculum, this could modify the findings at these schools. Though the educator survey sampled from a group of clinical skills and pediatric clerkship educators across the United States and Canada, response rate varied and there were not mechanisms to compare responses by region or within institutions.

## Conclusions

Our study confirms that students feel less prepared for their clinical experiences in pediatrics, in particular, pediatric physical exam skills, compared to their other clerkship specialties. It also demonstrates that clinical educators in clinical skills courses and pediatric clerkships both believe students should have some level of competence in pediatric physical exam skills prior to starting their pediatric clerkship. Medical schools should provide learning opportunities in a variety of healthcare settings that enable students to achieve specified pre-clerkship objectives. As medical schools move towards curriculum reform including optimization of earlier clinical education, we would suggest that pediatric content and skills development be included in the “pre-clinical” curriculum.

## Supplementary Information

**Additional file 1.** Pediatric and clinical skills educator physical exam survey.

## Data Availability

All data generated or analyzed during this study are included in this published article (and its supplementary information files).
