# Supplementary material for: Needs assessment for enhancing pediatric clerkship readiness
Source: BMC Med Educ. 2023 Mar 28;23:188. doi: 10.1186/s12909-023-04167-7 (PMC10044806; doi:10.1186/s12909-023-04167-7)
Supplement: Supplementary file 1 — Additional file 1. Pediatric and clinical skills educator physical exam survey. [file 12909_2023_4167_MOESM1_ESM.docx]

Pediatric and Clinical Skills Educator Physical Exam Survey

Question 1: Please identify your role in pediatric or clinical skills education

- Pediatrics Clerkship Director (including co-directors, assistant, and associate pediatric clerkship directors)
- Clinical Skills Course Director (including co-directors, assistant, and associate directors
- Other Course or Curricular Director in the Pre-Clinical Phase (including foundational science course directors or other longitudinal curriculum directors)
- Other

Question 2:


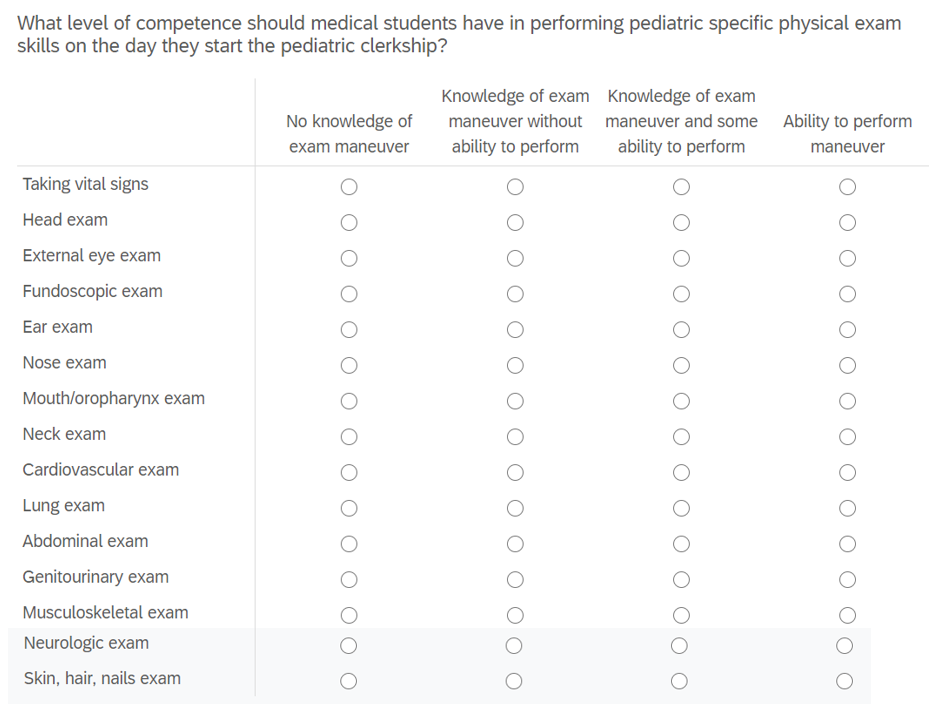


Question 3: What level of competence should medical students have in performing newborn specific physical exam skills on the day they start the pediatric clerkship?

- Newborn physical exam skills are not expected before clerkship
- Knowledge of newborn physical exam skills without ability to perform
- Knowledge of newborn physical exam skills with some ability to perform
- Ability to perform newborn physical exam

Question 4: What level of competence should medical students have in performing developmental assessment skills on the day they start the pediatric clerkship?

- Developmental assessment skills are not expected before clerkship
- Knowledge of developmental assessment skills without ability to perform
- Knowledge of developmental assessment skills with some ability to perform
- Ability to perform developmental assessment

Question 5: What other pediatric specific physical exam skills not listed above should a medical student be able to perform on the day they begin their pediatric clerkship?

Question 6: When **ARE** pediatric specific exam skills taught for all students in your school? (check all that apply)

- Longitudinal clinical skills course
- Transition time before beginning any clerkships
- Orientation at start of pediatric clerkship
- During the pediatric clerkship in a formal physical exam skills session
- During the pediatric clerkship through experiential learning with patients and preceptors
- Other

Question 7: When do you believe pediatric specific exam skills **SHOULD** be taught for all students in your school? (check all that apply)

- Longitudinal clinical skills course
- Transition time before beginning any clerkships
- Orientation at start of pediatric clerkship
- During the pediatric clerkship in a formal physical exam skills session
- During the pediatric clerkship through experiential learning with patients and preceptors
- Other
